# Anhedonia, Apathy, Pleasure, and Effort-Based Decision-Making in Adult and Adolescent Cannabis Users and Controls

**DOI:** 10.1093/ijnp/pyac056

**Published:** 2022-08-24

**Authors:** Martine Skumlien, Claire Mokrysz, Tom P Freeman, Vincent Valton, Matthew B Wall, Michael Bloomfield, Rachel Lees, Anna Borissova, Kat Petrilli, Manuela Giugliano, Denisa Clisu, Christelle Langley, Barbara J Sahakian, H Valerie Curran, Will Lawn

**Affiliations:** Department of Psychiatry, University of Cambridge, Cambridge, UK; Clinical Psychopharmacology Unit, Clinical Educational and Health Psychology Department, University College London, London, UK; Clinical Psychopharmacology Unit, Clinical Educational and Health Psychology Department, University College London, London, UK; Clinical Psychopharmacology Unit, Clinical Educational and Health Psychology Department, University College London, London, UK; Institute of Cognitive Neuroscience, Division of Psychology and Language Sciences, University College London, London, UK; Clinical Psychopharmacology Unit, Clinical Educational and Health Psychology Department, University College London, London, UK; Division of Psychiatry, University College London, London, UK; Clinical Psychopharmacology Unit, Clinical Educational and Health Psychology Department, University College London, London, UK; Addiction and Mental Health Group (AIM), Department of Psychology, University of Bath, Bath, UK; Clinical Psychopharmacology Unit, Clinical Educational and Health Psychology Department, University College London, London, UK; Institute of Psychiatry, Psychology and Neuroscience, King’s College London, London, UK; Clinical Psychopharmacology Unit, Clinical Educational and Health Psychology Department, University College London, London, UK; Addiction and Mental Health Group (AIM), Department of Psychology, University of Bath, Bath, UK; Clinical Psychopharmacology Unit, Clinical Educational and Health Psychology Department, University College London, London, UK; Clinical Psychopharmacology Unit, Clinical Educational and Health Psychology Department, University College London, London, UK; Department of Psychiatry, University of Cambridge, Cambridge, UK; Behavioural and Clinical Neuroscience Institute, University of Cambridge, Cambridge, UK; Department of Psychiatry, University of Cambridge, Cambridge, UK; Behavioural and Clinical Neuroscience Institute, University of Cambridge, Cambridge, UK; Clinical Psychopharmacology Unit, Clinical Educational and Health Psychology Department, University College London, London, UK; Clinical Psychopharmacology Unit, Clinical Educational and Health Psychology Department, University College London, London, UK; Department of Addictions, Institute of Psychiatry Psychology and Neuroscience, King’s College London, London, UK

**Keywords:** Cannabis, adolescent, reward, effort, motivation

## Abstract

**Background:**

Cannabis use may be linked with anhedonia and apathy. However, previous studies have shown mixed results, and few have examined the association between cannabis use and specific reward sub-processes. Adolescents may be more vulnerable than adults to harmful effects of cannabis. This study investigated (1) the association between non-acute cannabis use and apathy, anhedonia, pleasure, and effort-based decision-making for reward; and (2) whether these relationships were moderated by age group.

**Methods:**

We used data from the “CannTeen” study. Participants were 274 adult (26–29 years) and adolescent (16–17 years) cannabis users (1–7 d/wk use in the past 3 months) and gender- and age-matched controls. Anhedonia was measured with the Snaith-Hamilton Pleasure Scale (n = 274), and apathy was measured with the Apathy Evaluation Scale (n = 215). Effort-based decision-making for reward was measured with the Physical Effort task (n = 139), and subjective wanting and liking of rewards was measured with the novel Real Reward Pleasure task (n = 137).

**Results:**

Controls had higher levels of anhedonia than cannabis users (*F*_1,258_ = 5.35, *P* = .02, η _p_^2^ = .02). There were no other significant effects of user-group and no significant user-group*age-group interactions. Null findings were supported by post hoc Bayesian analyses.

**Conclusion:**

Our results suggest that cannabis use at a frequency of 3 to 4 d/wk is not associated with apathy, effort-based decision-making for reward, reward wanting, or reward liking in adults or adolescents. Cannabis users had lower anhedonia than controls, albeit at a small effect size. These findings are not consistent with the hypothesis that non-acute cannabis use is associated with amotivation.

**Significance Statement**

Cannabis use has historically been linked with amotivation, which is reflected in prevalent, pejorative “lazy stoner” stereotypes. In this study, we counter this cliché by showing that a relatively large group of adult and adolescent cannabis users and controls did not differ on several measures of reward and motivation. Specifically, people who used cannabis on average 4 d/wk did not report greater apathy or anhedonia, reduced willingness to expend effort for reward, or reduced reward wanting or liking compared with people who did not use cannabis. Additionally, while adolescents had greater apathy and anhedonia than adults, cannabis use did not augment this difference; thus, adolescents were not more sensitive to the putatively damaging effect of cannabis. Our results add to the growing body of evidence suggesting that non-acute cannabis use is not linked with amotivation, which may help to reduce stigma experienced by people who use cannabis.

## Introduction

Cannabis is the third-most commonly used controlled substance worldwide after alcohol and nicotine (United Nations Office on Drugs and Crime, 2020). In the 2020 European Drug Report (EMCDDA, 2020), 19% of 15- to 24-year-olds reported past-year cannabis use compared with 15% of 15- to 34-year-olds and 7.6% of 15- to 64-year-olds. Annual prevalence is estimated at 19.3% among 15-year-olds in England (NHS Digital Lifestyles Team, 2018), and 28.0% of 15- to 16-year-olds in the United States (National Institute on Drug Abuse, 2020). Thus, cannabis use is disproportionately high among adolescents. Adolescents may be particularly susceptible to effects of cannabis on mental health and cognition, including reward processing (Schneider, 2008).

Reward processing refers to any process that underpins the seeking and consumption of rewards (Berridge and Robinson, 2016) and encompasses several reward sub-processes (Berridge et al., 2009; Husain and Roiser, 2018). Syndromes of disrupted reward processing include apathy, defined as a loss of or reduction in motivation (Robert et al., 2009), and anhedonia, defined as a loss of interest in or pleasure from previously rewarding activities (Treadway and Zald, 2011). The endocannabinoid system plays a central role in brain reward processes, chiefly through modulation of dopaminergic and opioidergic neurotransmission (Solinas et al., 2008; Wenzel and Cheer, 2018). Cannabis acts on the endocannabinoid system, and repeated exposure may impair its sensitivity to rewarding stimuli and increase the susceptibility to anhedonia and apathy in cannabis users (Volkow et al., 2017). In this study, we simultaneously assessed multiple reward sub-processes to gain a better understanding of the relationship between cannabis use and reward.

Prevalent, derogatory “stoner” stereotypes portray cannabis users as lazy and demotivated (McGlothlin and West, 1968; Mortensen et al., 2020); however, limited scientific evidence exists to support this claim. In a recent systematic review, we found only 2 studies comparing behavioral motivation in cannabis users and controls, operationalized as willingness to expend effort for reward (Skumlien et al., 2021b). Lane et al. (2005) found lower motivation in 14 adolescent cannabis users compared with 20 controls, whereas Lawn et al. (2016) did not find a similar effect in 40 adult users and controls. More recently, and using larger samples of 86 participants and 60 participants, respectively, both Taylor and Filbey (2021) and Vele et al. (2022) found that adult cannabis users selected hard trials on the Effort Expenditure for Reward task (EEfRT) more often than adult controls. Similarly, Acuff et al. (2022) found that frequency of cannabis use and symptoms of cannabis use disorder were positively associated with selecting a high-effort trial in a sample of 47 young adult cannabis users and controls.

The same systematic review also found some evidence of an association between cannabis use and apathy. However, results were inconsistent. For instance, 1 recent cross-sectional study of 1168 young adults found that apathy, assessed with the Apathy Evaluation Scale (AES), correlated positively with quantity of cannabis use and problematic use, but not with frequency of use or age of onset (Petrucci et al., 2020). However, effect sizes were small, with the largest correlation at *r* = .125 when accounting for depression, other substance use, and personality characteristics. No significant relationship was found in another large study of 487 adults (Barnwell et al., 2006) or in a recent longitudinal study of 401 adolescents (Pacheco-Colón et al., 2021), both using the AES. There was stronger evidence supporting an association between cannabis use and anhedonia in adolescents (Skumlien et al., 2021b). One large and longitudinal study by Leventhal et al. (2017) (n = 3394), which adjusted for mental health variables and polysubstance use, found that anhedonia at age 14 predicted future cannabis use but not vice versa. Anhedonia was measured with the Snaith-Hamilton Pleasure Scale (SHAPS) in this study.

Adolescence is an important period of socio-emotional, cognitive, and brain development, during which external factors such as cannabis and other substance use may be particularly influential in shaping the brain and cognition (Giedd et al., 1999; Giedd, 2004; Schneider, 2008; Bossong and Niesink, 2010; Lubman et al., 2015). Grey matter differences between adults and adolescents are pronounced in frontal and striatal regions (Sowell et al., 1999), which are important to reward and motivation (Oldham et al., 2018), and adolescents may overactivate limbic and striatal regions during reward-processing tasks (Galvan et al., 2006; Silverman et al., 2015). Adolescence is also an important period for maturation of the endocannabinoid system, which plays a central role in several neurodevelopmental processes, including neural proliferation, differentiation, and migration (Harkany et al., 2008; Viveros et al., 2012). Therefore, adolescents may be more vulnerable to the presumed disruptive effects of cannabis on reward processing compared with adults. Consistent with this, we recently found that adolescents were more susceptible to cannabis-related anhedonia on the SHAPS than adults, with adolescent dependent users showing the highest levels of anhedonia and apathy overall (Skumlien et al., 2021a).

There are multiple gaps in the existing literature. First, relatively few studies have examined specific reward sub-processes concomitantly, including effort-related decision-making and pleasure taken from real rewards. Behavioral tasks are valuable for assessing specific components of reward processing that may be affected in apathy and anhedonia (Husain and Roiser, 2018). Additionally, previous studies using task-based measures of reward and motivation in cannabis users have typically suffered from small sample sizes. Finally, despite the hypothesized adolescent vulnerability to harmful effects, there are remarkably few studies comparing current adult and adolescent cannabis users directly on cognitive or psychological outcomes. In the current study, we address these gaps by comparing a relatively large sample of adult and adolescent cannabis users, matched on cannabis use frequency, and age-matched controls on 2 novel tasks assessing effort-based decision-making and subjective explicit reward wanting and liking as well as questionnaire assessments of anhedonia and apathy. We propose the following, pre-registered (Skumlien et al., 2020) hypotheses:

1. Cannabis users will have higher levels of anhedonia and apathy compared with controls.
2. Cannabis users will show lower willingness to expend effort for reward and lower subjective reward wanting and liking compared with controls.
3. There will be interactions between user-group and age-group for all outcomes, whereby differences will be larger between adolescent users and age-matched controls than between adult users and age-matched controls.

## Methods

### Study Design

The current study presents cross-sectional, baseline data from the longitudinal arm of the CannTeen study (Lawn et al., 2020). The study has 2 between-subjects factors: age-group (adolescents and adults) and user-group (users and controls).

### Participants

Participants were 76 adolescent cannabis users, 63 adolescent controls, 71 adult cannabis users, and 64 adult controls, recruited from the Greater London area via school assemblies, physical posters and flyers, and social media advertisements. Adults were 26–29 years of age, and adolescents were 16–17 years of age. The full sample of 274 participants completed the anhedonia questionnaire measure, and 215 participants completed the apathy questionnaire measure. A sub-sample of 139 participants (34 adolescent users, 35 in each remaining group) completed the task-based measures.

The key inclusion criterion for cannabis users was having used cannabis 1–7 d/wk, on average, over the past 3 months. Adult users were excluded if they had used cannabis regularly prior to the age of 18 to isolate the impact of adolescent cannabis use. Key inclusion criteria for controls were having used cannabis or tobacco at least once but having <10 lifetime uses of cannabis, and having no cannabis use in the month prior to the baseline session. Exclusion criteria for all participants were use of any psychotropic medication on a daily basis, past-month treatment for a mental health condition (including cannabis dependence), and use of any one illicit drug more than twice per month over the past 3 months. Full inclusion and exclusion criteria are detailed in supplemental Table 1 and the study protocol (Lawn et al., 2020). All participants provided written and informed consent to participating. The study was approved by the University College London ethics committee, project ID 5929/003 and conducted in line with the Declaration of Helsinki.

### Materials

#### Questionnaire Measures

Anhedonia was assessed with the SHAPS (Snaith et al., 1995), and apathy was assessed with the AES (Marin et al., 1991). Both measures have been demonstrated as reliable and valid in both adults (Franken et al., 2007; Raimo et al., 2014; Lueken et al., 2017) and adolescents (Leventhal et al., 2015; Pacheco-Colón et al., 2018).

Higher scores indicated higher levels of anhedonia and apathy, respectively. Additional details are in the supplemental Methods.

#### Behavioral Tasks

Behavioral measures were the Physical Effort task (PhEft) and the Real Reward Pleasure task (RRPt). Full details are in the supplemental Methods.

The PhEft was developed based on the EEfRT (Treadway et al., 2009; Husain and Roiser, 2018), and similar versions have been used in previous studies (Bonnelle et al., 2016; Valton et al., 2017). Participants were given the option to perform button-presses to win points, which were later exchanged for chocolates or sweets. There were 3 difficulty levels and 3 reward levels, which were presented at the beginning of each trial. The participant could choose to accept or reject the offer, and the number of acceptances indicated the participants’ overall willingness to expend effort for reward. Additionally, reward sensitivity scores were computed by subtracting the number of accepted trials at the lowest reward level from the number of accepted trials at the highest reward level. Effort sensitivity scores were computed by subtracting the number of accepted trials at the highest effort level from the number of accepted trials at the lowest effort level. These were used to indicate the participants’ sensitivity to changes in reward magnitude and effort requirement, respectively, with higher scores indicating greater sensitivity. Supplemental Figure 1 provides a diagram of the task.

The RRPt was developed in previous studies (Lawn et al., 2015, 2018) and mimics existing reward liking tasks that have been validated in cannabis users and other populations (Berridge et al., 2009; Ford et al., 2014; de Bruijn et al., 2017; Freeman et al., 2018). Participants were first told to estimate how much they wanted to receive each of 3 rewards (30 seconds of 1 of their favorite songs, 1 piece of chocolate/candy, and a 1-pound coin). They then received each reward in turn and were asked to rate how pleasurable they found them. Ratings were averaged across the type of reward to produce mean reward wanting and mean reward liking scores for each participant.

#### Covariates

Covariates were depression, risk-taking, and maternal education plus frequency of alcohol, tobacco, and other illicit drug use. These were chosen a priori due to their possible interaction with cannabis use and reward processing (Patton et al., 2002; Fergusson et al., 2006; Balodis and Potenza, 2015; Leadbeater et al., 2019; Millar et al., 2021). All drug use was assessed with the timeline follow-back (Robinson et al., 2014). Additional details are in the supplemental Methods.

### Procedure

Data collection procedures are presented in full in the CannTeen study protocol (Lawn et al., 2020). Demographic, drug use, and questionnaire data were collected at a baseline behavioral session. The PhEft and RRPt were completed at a baseline neuroimaging session, which was typically conducted within 2 weeks, and always within 2 months, of the behavioral session. Tasks were completed outside the scanner in a quiet room at the imaging center. Neuroimaging results are presented elsewhere (Skumlien et al., 2022). Participants completed an instant saliva drug test, a breathalyzer, and self-reported abstinence to confirm no recent use of alcohol or cannabis (≥12 hour cut-off) or illicit drugs (≥48 hour cut-off) at the start of all study sessions. Participants with a BAC > 0 or positive result for or self-report of recent use of any illicit drug (including cannabis/Δ ^9^-tetrahydrocannabinol) were rescheduled.

### Analyses

Analyses and hypotheses were pre-registered on the Open Science Framework (Skumlien et al., 2020). Analyses were performed in R 3.6.2 (R Core Team, 2019), with the rstatix package (Kassambara, 2021) and BayesianFactor package (Morey and Rouder, 2018). All data were inspected to ensure the assumptions of parametric statistics were met.

#### Missing Data

Due to experimenter error, item 4 of the AES was omitted and missing for all participants. This was imputed using the participant-level means of the cognitive subscale rounded to the nearest integer. Other missing items were imputed using the mean of the relevant subscale for AES, and the mean score from the full questionnaire for the SHAPS, rounded to the nearest integer. Participants with reward or effort sensitivity scores ≤0 were omitted from the relevant analysis. This was to exclude participants who may not have performed the task correctly and to avoid zero-inflation. Supplemental Table 2 gives an overview of missing and imputed items and exclusions.

#### Statistical Models

Internal consistency for the SHAPS and AES was assessed with Cronbach’s alpha. All outcomes (SHAPS, AES, PhEft total acceptances, PhEft reward sensitivity, PhEft effort sensitivity, RRPt reward wanting, RRPt reward liking) were analyzed with 2 × 2 analyses of covariance (ANCOVAs), with factors user-group, age-group, and their interaction. An additional ANCOVA was performed for the truncated AES, excluding the imputed item 4, as a sensitivity analysis. Covariates were included as specified in the “covariates” section. Null results were followed-up with post-hoc Bayesian independent-samples *t* tests for cannabis users compared with controls and adult users compared with adolescent users. This was because Bayesian tests can quantify evidence for the null hypothesis. A scaled-information prior of *r* = .707 was used, and Jeffreys-Zellner-Siow Bayes factors (BF_01_) ≥3 were interpreted as meaningful and supportive of the null hypothesis (Wagenmakers et al., 2011). We also computed bivariate correlations between all reward processing outcomes and cannabis use frequency (days/week of use in the past 3 months). Finally, we computed exploratory bivariate correlations between all reward processing outcomes to better understand the interrelationships between reward processing measures.

## Results

**Figure 1.**
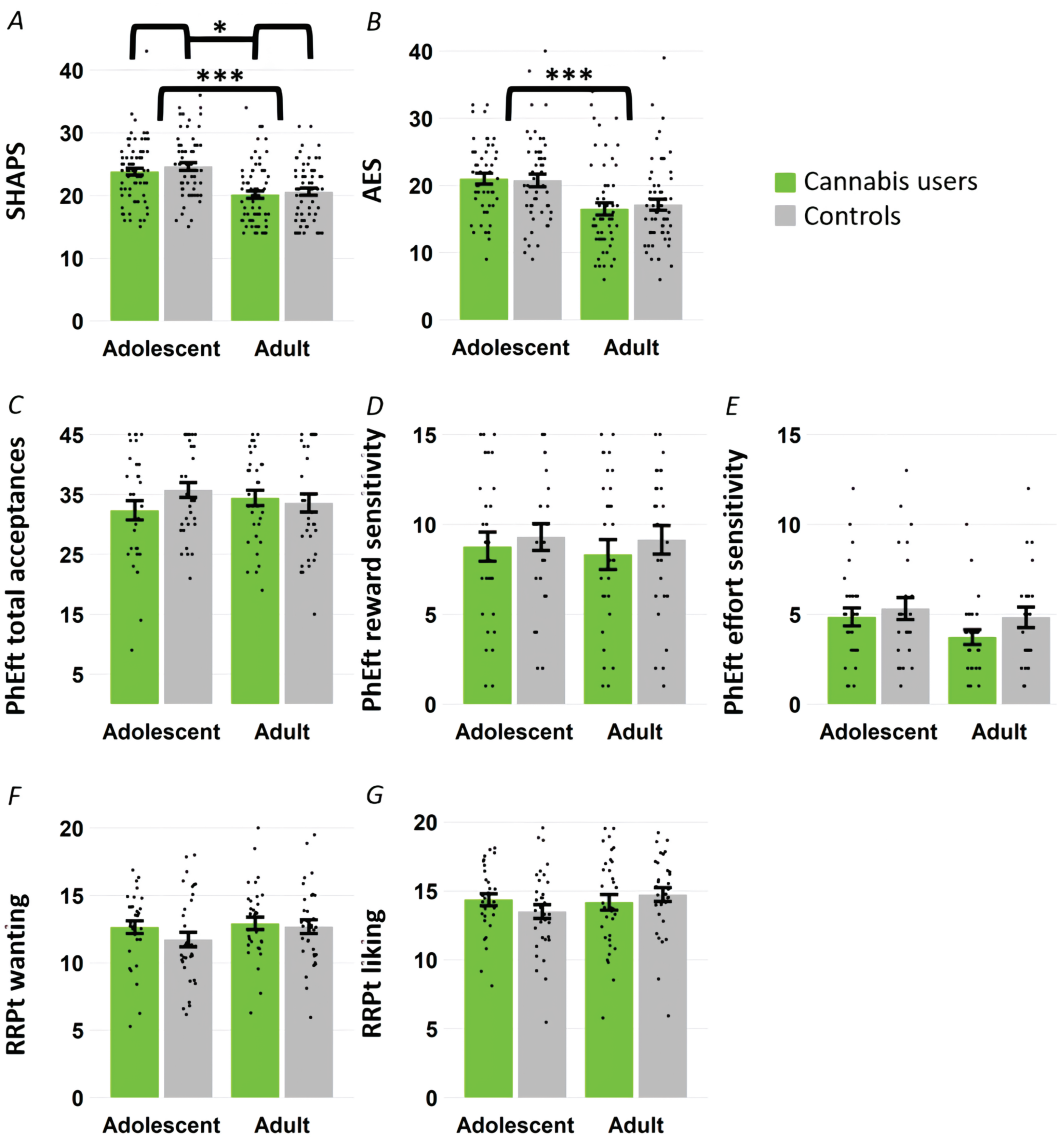
Group differences in all reward processing outcomes. Bars represent means, dots indicate individual participant values, and error bars represent standard errors. (A) Snaith-Hamilton Pleasure Scale, n = 268. Higher scores indicate higher levels of anhedonia. A 2 × 2 analysis of covariance controlling for depression, risk-taking, maternal education, and alcohol, tobacco, and other drug use showed significantly greater anhedonia in controls than cannabis users (*P* = .02) and in adolescents than adults (*P* < .001). (B) Apathy Evaluation Scale, n = 211. Higher scores indicate higher levels of apathy. A 2 × 2 analysis of covariance controlling for depression, risk-taking, maternal education, and alcohol, tobacco, and other drug use, showed a significant difference between adults and adolescents (*P* < .001). (C) Physical effort task total acceptances, n = 137. (D) Physical effort task reward sensitivity, n = 112. (E) Physical effort task effort sensitivity, n = 103. (F) Real reward pleasure task reward wanting, n = 135. (G) Real reward pleasure task reward liking, n = 135.

Results of all models are displayed in Figure 1.

Group sizes for each task and reasons for exclusion are reported in supplemental Table 2. Sample characteristics for the full sample are reported in Tables 1 and 2 and for the sub-sample in supplemental Tables 3 and 4. Finally, correlations between reward processing outcomes are presented in supplemental Table 5. There was a strong and significant correlation between scores on the SHAPS and AES (*r* = .515, *P* < .001) and between RRPt reward liking and SHAPS (*r* = −.288, *P* < .001) and AES scores (*r* = −.244, *P* = .004). PhEft effort sensitivity also correlated with AES scores (*r* = .210, *P* = .03), but this was not significant after correction for multiple comparisons.

**Table 1.** Sample Characteristics for the Full Sample

	Adolescent users (n = 75)	Adult users (n = 68)	Adolescent controls (n = 62)	Adult controls (n = 63)	Group differences
Gender					
Female	38 (51%)	30 (44%)	32 (52%)	33 (52%)	ns
Male	37 (49%)	38 (56%)	30 (48%)	30 (48%)	
Age, y	17.09 (0.50), 16.26–18.0	27.63 (1.14), 26.0–29.92	17.12 (0.47), 16.05–18.0	27.41 (1.01), 26.01–29.91	Adults > Adolescents^a^
Ethnicity, n (%)					
White	51 (68%)	43 (63%)	39 (63%)	41 (65%)	
Mixed	15 (20%)	8 (12%)	7 (11%)	3 (5%)	
Asian	2 (3%)	10 (15%)	10 (16%)	14 (22%)	
Black	4 (5%)	6 (9%)	2 (3%)	2 (3%)	
Other	3 (4%)	1 (1.5%)	2 (3%)	2 (3%)	
Prefer not to say	0 (0%)	0 (0%)	2 (3%)	1 (2%)	
Maternal education					
Below undergraduate degree	31 (41%)	37 (54%)	26 (42%)	36 (57%)	Adolescents > Adults^b^
Undergraduate degree or above	44 (59%)	31 (46%)	36 (58%)	27 (43%)	
BDI	12.77 (8.38), 1–41	7.75 (8.60), 0–46	10.03 (7.60), 0–40	6.97 (6.58), 0–28	Adolescents > Adults^a^
RT-18	11.39 (3.10), 3–18	8.63 (3.91), 3–17	9.11 (4.11), 0–17	7.65 (4.04), 0–16	Users > Controls^a^ Adolescents > Adults^a^
WTAR	111.81 (9.04), 90–127, n = 73	107.25 (9.95), 85–124, n = 67	110.71 (10.43), 85–126	110.54 (9.64), 85–124	Adolescents > Adults^b^
Alcohol use, d/wk	0.63 (0.64), 0–3.25	1.46 (1.42), 0–6.83	0.67 (0.76), 0–3.67	1.46 (1.05), 0–5.25	Adults > Adolescents^a^
Cigarette/roll-up use, d/wk	2.24 (2.62), 0–7	1.35 (2.51), 0–7	0.52 (1.57), 0–6.58	0.45 (1.40), 0–7	Users > Controls^a^
Other illicit drug use, monthly use					Users > Controls^a^
Yes	44 (59%)	17 (25%)	2 (3.23%)	1 (2%)	Adolescents > Adults^a^
No	31 (41%)	51 (75%)	60 (97%)	62 (98%)	

Abbreviations: BDI, Beck Depression Inventory; RT-18, Risk-taking 18; WTAR, Wechsler Test of Adult Reading.

Sample characteristics are displayed for n = 268, which is the maximum number of participants from the full sample included in at least 1 analysis. For continuous data mean (SD) and range are shown. For categorical data, n (%) is shown. Group differences were investigated with 2 × 2 analyses of variance, independent samples *t* tests, or chi-square tests of independence. All variables were assessed at the baseline behavioral session. The WTAR was used as a measure of premorbid intelligence (Wechsler, 2001).

^a^ *P* <.001,

^b^ *P* < .05.

**Table 2.** Cannabis Use Variables for the Full Sample

	Adolescent users (n = 75)	Adult users (n = 68)	Adolescent controls (n = 62)	Adult controls (n = 63)	Group differences
Ever use (controls)			54 (87%)	61 (97%)	ns
No. of lifetime uses (controls)			3.42 (2.84), 0–10	4.52 (3.08), 0–10	Adults > adolescents^a^
Days/week of use (users)	3.74 (1.97), 0.83–6.92	4.17 (1.90), 0.75–6.92			ns
No. of users who most commonly use strong herbal cannabis (i.e., “skunk”)	68 (91%)	57 (84%)			ns
Grams used on day of use (users)	1.05 (0.82), 0.08–4	0.62 (0.66), 0.03–3.5, *n* = 65			Adolescents > adults^b^
Days since last use (users)	2.41 (2.59), 0.54–14	2.56 (4.70), 0.5–35			ns
Age of first ever use (users)	14.61 (1.15), 11.0–16.58	18.01 (2.94), 13.0–25.0			Adults > adolescents^b^
Age of first weekly use (users)	15.65 (1.02), 13.0–17.67	22.25 (2.78), 17.0–27.67			Adults > adolescents^b^
CUDIT-R (users)	15.40 (5.59), 5–27	11.87 (4.92), 3–26			Adolescents > adults^b^

Abbreviations: CUDIT-R, Cannabis Use Disorder Identification Test–Revised; ns, not significant.

Sample characteristics are displayed for n = 268, which is the maximum number of participants from the full sample included in at least 1 analysis. For continuous data mean (SD) and range are shown. For categorical data, n (%) is shown. Group differences were investigated with independent samples *t* tests or chi-square tests of independence. All variables were assessed at the baseline behavioral session.

^a^ *P* < .05,

^b^ *P* <.001.

### Anhedonia and Apathy

Full results for SHAPS and AES are presented in supplemental Table 6. Both the SHAPS and AES had good internal consistency, with Cronbach’s alpha values of .83 and.75, respectively. The SHAPS model yielded a significant effect of user-group (*F*_1,258_ = 5.35, *P* = .02, η _p_^2^ = .02) and age-group (*F*_1,258_ = 17.98, *P* < .001, η _p_^2^ = .065), but not their interaction (*F*_1,258_ = 1.01, *P* = .32, η _p_^2^ = .004). The unadjusted mean difference was 0.55 points between controls and users, and 3.84 points between adolescents and adults, indicative of higher anhedonia in controls and adolescents (see Figure 1). There was no correlation between anhedonia and cannabis use frequency (*r* = .07, *P* = .40).

AES subscale scores by group are displayed in supplemental Figure 2. The AES model yielded a significant effect of age-group (*F*_1,201_ = 13.89, *P* < .001, η _p_^2^ = .065), with adolescents scoring 4.05 points higher than adults. The effects of user-group and user-group*age-group were not significant (main *F*_1,201_ = 0.05, *P* = .82, η _p_^2 < ^.001; interaction *F*_1,201_ = 0.39, *P* = .54, η _p_^2^ = .002). Results remained the same when the analyses were re-run using only 17 AES items, excluding the imputed item 4. There was no correlation between apathy and cannabis use frequency (*r* = .16, *P* = .10). Bayesian analyses showed substantial evidence for the null hypothesis of no difference between users and controls on the AES (BF_01_ = 6.48).

### Physical Effort Task

Most participants had non-negative reward and effort sensitivity scores on the PhEft, indicating that the task had worked as expected (supplemental Table 2). There were no significant effects of user-group, age-group, or their interaction for total acceptances, reward sensitivity, or effort sensitivity. Frequency of use also did not correlate with total acceptances (*r* = −.01, *P* = .93), reward sensitivity (*r* = .06, *P* = .65), or effort sensitivity (*r* = .03, *P* = .85). Bayesian analyses yielded substantial evidence for the null hypothesis of no difference between cannabis users and controls for acceptances (BF_01_ = 3.78) and reward sensitivity (BF_01_ = 3.58) but not effort sensitivity (BF_01_ = 1.89). There was also substantial evidence for the null hypothesis of no difference between adult and adolescent users for reward sensitivity (BF_01_ = 3.58), but not for acceptances (BF_01_ = 2.60) or effort sensitivity (BF_01_ = 1.09). Full results are presented in supplemental Table 7.

### Real Reward Pleasure Task

All but 1 participant rated all rewards greater than zero on the RRPt, indicating that the task had worked as expected. There were no significant effects of user-group, age-group, or their interaction for RRPt wanting or liking. Frequency of use also did not correlate with reward wanting (*r* = −.18, *P* = .15) or reward liking (*r* = −.18, *P* = .14). Bayesian analyses supported the null hypothesis of no difference between users and controls for reward liking (BF_01_ = 5.16) but not for reward wanting (BF_01_ = 2.78). Bayesian analyses also supported the null hypothesis of no difference between adult users and adolescent users for both reward wanting (BF_01_ = 3.69) and liking (BF_01_ = 3.87). Full results can be found in supplemental Table 8. Mean wanting and liking ratings for each reward type are displayed in supplemental Table 9.

## Discussion

In the current study, we compared adult and adolescent cannabis users, matched on cannabis frequency, with gender- and age-matched controls on several reward processing measures. Cannabis users had significantly lower levels of anhedonia than controls by roughly one-half a point on the SHAPS, and adolescents had significantly higher levels of both anhedonia and apathy than adults by roughly 4 points on both the SHAPS and AES, respectively. There were no significant main or interaction effects for willingness to expend effort for reward, reward sensitivity, effort sensitivity, reward wanting, or reward liking. Null findings were broadly supported by Bayesian analyses. In summary, the hypothesis that non-acute cannabis use is associated with reward processing impairments was not supported.

### Anhedonia and Apathy

The current finding of lower anhedonia in cannabis users was contrary to our hypotheses. It could be that cannabis potentiates the reinforcing effects of some rewards (e.g., Solinas et al., 2008) or that people who are more prone to seek out pleasure are also more likely to use cannabis. However, the mean difference between cannabis users and controls was <1 point on the SHAPS, corresponding to a small effect size (η _p_^2^ = .02). In comparison, Franken et al. (2007) found a 14-point difference between healthy controls and people with depression. This finding may therefore not be clinically relevant and should be interpreted with caution.

Previous well-controlled studies using large samples have found a positive relationship between cannabis use and anhedonia in adolescents (Leventhal et al., 2017) but not adults (Skumlien et al., 2021a). In fact, Skumlien et al. (2021a) found a negative association between cannabis use and anhedonia in adults after the coronavirus lockdown, consistent with the present results. The largest study to date found significant and positive, albeit weak associations between apathy, quantity of use, and problematic use (Petrucci et al., 2020), incongruent with the present findings. However, consistent with Petrucci et al. (2020), we did not find a significant correlation with frequency of use. Moreover, our results converge with a number of other large-scale studies of apathy in cannabis users, which have yielded null results (Barnwell et al., 2006; Pacheco-Colón et al., 2021; Skumlien et al., 2021a).

It is possible that group differences would have emerged with more frequent or problematic cannabis use in the user group. However, participants used cannabis on average 4 d/wk, which is similar to previous studies that have found significant cannabis effects (e.g., Lopez-Vergara et al., 2019; Skumlien et al., 2021a), and frequency of use did not correlate with apathy or anhedonia. Furthermore, mean scores on the Cannabis Use Disorder Identification Test were high, with 56 adolescents (74.7%) and 33 adults (48.5%) meeting the cut-off for at least mild cannabis use disorder (Adamson et al., 2010). Still, the distinction between daily use of large quantities and non-daily cannabis use is important. For instance, it could be that cannabis has acute amotivational or anhedonic effects (Lawn et al., 2016; Wardle et al., 2022), which may result in a persistent apathetic or anhedonic state if used daily, disregarding any tolerance effects. Duration of abstinence in the present study was at least 12 hours and typically 2 days, minimizing residual effects of acute intoxication.

The relationship between cannabis, anhedonia, and apathy is likely to be complex, and the interpretation of previous results is complicated by lack of ability to assess causality as well as potential confounding and/or moderating variables. For instance, Leventhal et al. (2017) found that anhedonia positively predicted cannabis use, rather than the other way around. Additionally, cannabis might have indirect effects on apathy and anhedonia by increasing the risk of psychosis and depression (Moore et al., 2007; Lev-Ran et al., 2014). Finally, it is worth noting that self- and observer ratings may differ. Popular beliefs about how cannabis affects motivation might engender a biased perception of users as less motivated than they actually are. Meier and White (2018) is the only study to have looked at informant-reported apathy and found that cannabis users were rated as significantly more apathetic than controls, which contrasts with the null findings reported in the present study of self-reported apathy. It could also be that cannabis users perceive that other people (e.g., the researcher) view them as demotivated, which might prompt a desire to appear more motivated in psychological studies, possibly biasing the present results. Future comparisons of self-rated and observer-rated anhedonia and apathy in cannabis users would be informative.

### Behavioral Tasks

Contrary to our hypotheses, there were no main or interaction effects for any outcomes on the physical effort task or the real reward pleasure task. There was a significant and negative effect of the depression covariate on PhEft total acceptances and reward sensitivity (supplementary Table 7), and RRPt reward liking (supplementary Table 8). This demonstrates the validity of the tasks, given the existing relationship between depression and compromised reward processing (Eshel and Roiser, 2010). Moreover, reward liking correlated negatively with both the SHAPS and AES (supplementary Table 5).

Previous studies using similar behavioral assessments of motivation have yielded mixed evidence for altered effort-based decision-making for reward in cannabis users, with the 3 most recent studies finding a positive association between cannabis use and willingness to expend effort for reward on the EEfRT (Taylor and Filbey, 2021; Acuff et al., 2022; Vele et al., 2022). Unlike the EEfRT, rewards in the PhEft are food-based and non-probabilistic, which could explain the difference in findings. Nonetheless, although motivation is a multi-faceted concept and additional studies using alternative measures are needed to comprehensively assess the potential link with cannabis use, present and previous evidence suggests that non-acute cannabis use is not associated with lower willingness to expend effort for reward.

There are only 2 existing studies, to the authors’ knowledge, that assess the association between cannabis use and some subjective measure of reward liking (Skumlien et al., 2021b). These showed lower mood responses to positive feedback on a spatial delayed response task (Martin-Soelch et al., 2009) and a lower increase in pleasantness ratings to female compared with male touch in cannabis users compared with controls (Zimmermann et al., 2019). However, their small sample sizes and relatively complex designs limit their ecological validity, and in both cases a significant difference between cannabis users and controls was found for only a few specific statistical comparisons or trial types. The RRPt has the advantage that it provides clear, in-the-moment assessment of responses to several typical rewards. Our results suggest that cannabis use is not associated with reduced subjective wanting or liking of food, money, and music rewards. However, future studies using alternative rewards, perhaps also in different quantities and settings, are needed to corroborate these findings. Moreover, as previously mentioned, it is possible that heavier use is associated with different effects.

### Age-Group Differences

Our results suggested that adolescents had higher anhedonia and apathy compared with adults but that cannabis use did not augment this difference. There was no indication of adolescent vulnerability to cannabis effects on effort-based decision-making, reward wanting, or reward liking. Importantly, adult, and adolescent cannabis users were matched on frequency of use and days since last use. Where they differed, it was in the direction of greater use quantity and levels of dependence as well as earlier age of onset in adolescent users compared with adult users. As such, lack of a significant interaction effect suggesting greater vulnerability in adolescents is unlikely to be due to different cannabis use patterns in the 2 age groups.

As previously discussed, some large-scale studies have found that anhedonia predicts cannabis use during adolescence (Leventhal et al., 2017) and that adolescent cannabis users are at greater risk of anhedonia than adult users (Skumlien et al., 2021a). Conversely, previous large-scale studies in adolescent samples have not found an association between cannabis use and apathy (Pacheco-Colón et al., 2021) or a greater risk of apathy in adolescent compared with adult users (Skumlien et al., 2021a), consistent with the present results. One previous study found evidence of reduced motivation/willingness to expend effort for reward in adolescent cannabis users compared with controls (Lane et al., 2005) but with a different task and smaller sample than the current study. Our study is the first, to our knowledge, to directly compare adolescent and adult cannabis users in the same study. Thus, our results, together with previous evidence, suggest that adolescents are not at a greater vulnerability to cannabis-related apathy, disrupted effort-based decision-making, or blunted reward wanting or liking compared with adults. However, longitudinal analyses are needed to confirm this.

Long-term and frequent cannabis use may still have related detrimental consequences in adolescents. Daily use may be associated with greater apathy due to greater duration of intoxication and could negatively impact educational achievement simply as a result of more time being spent using cannabis rather than on other activities. For instance, Pacheco-Colón et al. (2021) found a negative relationship between cannabis use and valuing of school in adolescents, and Schaefer et al. (2021) found that cannabis use was prospectively associated with decreased academic motivation during adolescence. Some functional neuroimaging studies have also found different neural reward processing responses in adolescent cannabis users compared with controls (Jager et al., 2013; Acheson et al., 2015; Nestor et al., 2020), though this has not been consistently found (Karoly et al., 2015), and there were no cannabis-related differences in the adolescent or adult reward system in a recent large-scale investigation from the CannTeen study (Skumlien et al., 2022). Still, adolescent cannabis use may be linked with other motivational outcomes that were not assessed in the present study.

In our current cross-sectional CannTeen analyses, the consistent lack of significant age-group by user-group interactions, supported by Bayesian analyses, is striking. We have also not found significant age-group by user-group interactions for depression, anxiety, or psychotic-like symptoms (Lawn et al., 2022b), or verbal episodic memory, spatial working memory, or response inhibition (Lawn et al., 2022a) using the same sample. Our results suggest that the adolescent reward system may not be vulnerable to substantial harm from non-acute cannabis at a moderate frequency of 4 d/wk. This could be because cannabis does not chronically compromise the reward system (Skumlien et al., 2022), perhaps because the reward system has matured enough by age 16 to not be sensitive to disruption (Casey et al., 2008). Alternatively, the impact of adolescent cannabis use on reward processing may be delayed and not seen until later in life.

### Strengths and Limitations

Strengths of this study include assessment of reward processing across multiple domains, pre-registration of analyses, rigorous assessment of cannabis and other drug use using the timeline follow-back, biological verification of recent abstinence, adjustment of relevant confounders, matching of adolescent and adult users for level of cannabis use, and the novel comparison of both adult and adolescent user groups with gender- and age-matched controls on reward processing outcomes.

An important limitation of the current study is the cross-sectional design. The impact of cannabis on reward processing in adolescence and young adulthood could have a time-lagged effect (e.g., Martz et al., 2016). Secondly, it is possible, albeit unlikely given our well-matched groups and adjustment for covariates, that pre-existing group differences obscured an effect of cannabis use. Thirdly, we purposely recruited cannabis users and matched controls to efficiently recruit frequent users; thus, our sample is not representative of the United Kingdom or the cannabis user population at large. Moreover, our sample was predominately White, albeit broadly like the UK population census. Fourth, to limit the risk of type 1 errors, we did not assess associations between reward processing outcomes and other measures of cannabis use, such as use quantity or cannabinoid content. Fifth, it is possible that younger or very frequent cannabis users show impairments in reward processing that we did not detect here. Finally, it is unclear whether the PhEft and RRPt generalize to real-life situations, and their formal reliability and validity have not yet been confirmed.

## Conclusions

Non-acute cannabis use at a moderate frequency of on average 4 d/wk was not linked with disrupted reward processing in either adults or adolescents over a range of domains. Adolescents were not at greater vulnerability to effects of cannabis on the assessed reward processing outcomes. In line with previous work (Lawn et al., 2016; Pacheco-Colón et al., 2021; Acuff et al., 2022), we argue that the collective evidence does not support an amotivational syndrome in cannabis users non-acutely, despite persistent “stoner” stereotypes. Future research should use longitudinal designs and diverse assessments of reward processing, examine ecological validity of reward measures, and investigate daily or near-daily users and even younger participants. A continued focus on adolescent users is warranted. Our findings should help to reduce stigma experienced by people who use cannabis by further dispelling claims of the “amotivational syndrome,” which increasingly appears lacking in scientific support.

## Supplementary Material

pyac056_suppl_Supplementary_Material

## Interest Statement

B.J.S. consults for Cambridge Cognition. H.V.C. has consulted for Janssen Research and Development. M.B.W.’s primary employer is Invicro LLC, a contract research organization which performs commercial research for the pharmaceutical and biotechnology industries. The remaining authors have no conflicts of interest to disclose.
