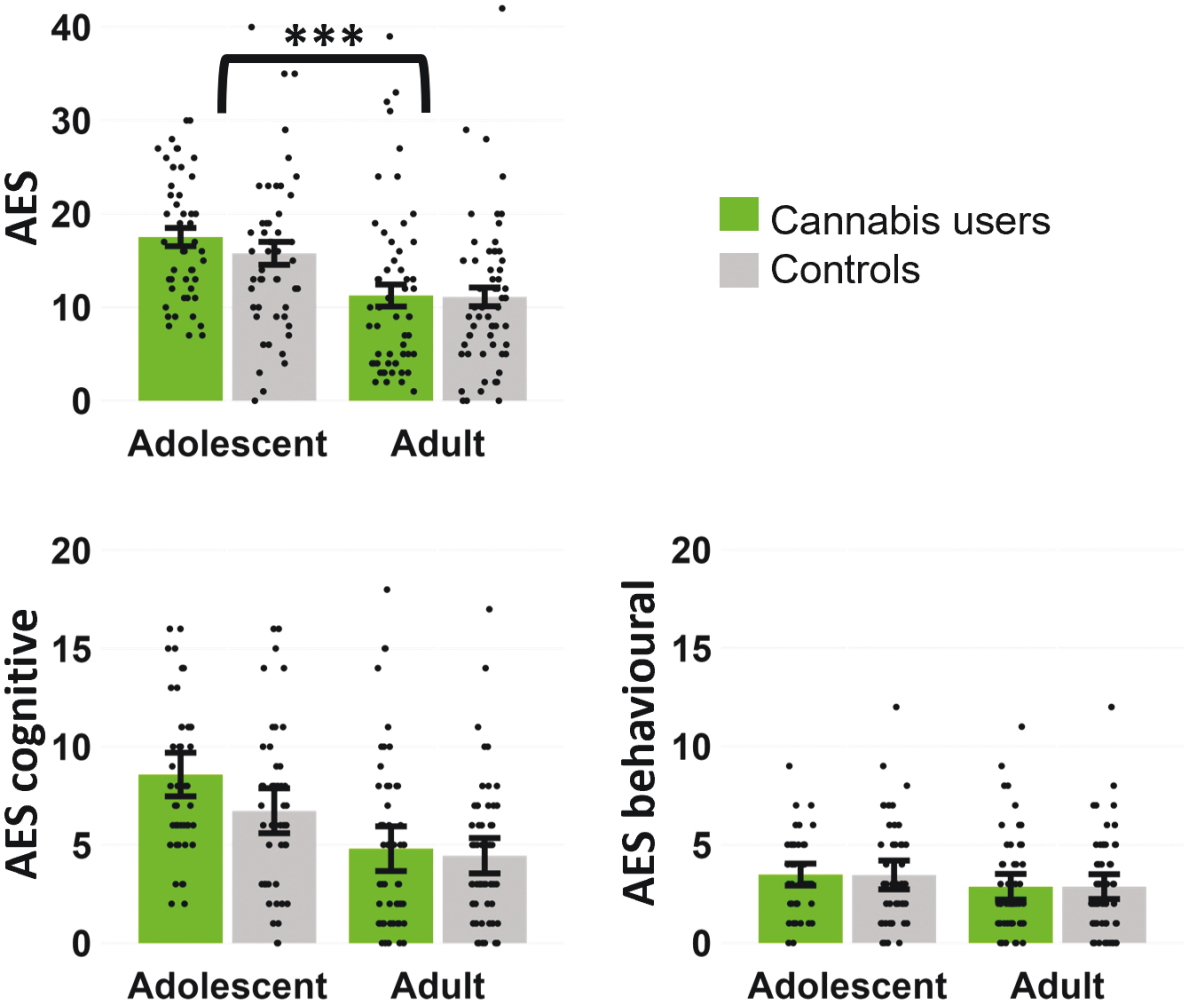# Corrigendum to: Anhedonia, Apathy, Pleasure, and Effort-Based Decision-Making in Adult and Adolescent Cannabis Users and Controls

**DOI:** 10.1093/ijnp/pyae004

**Published:** 2024-03-26

This is a corrigendum to: Martine Skumlien, Claire Mokrysz, Tom P Freeman, Vincent Valton, Matthew B Wall, Michael Bloomfield, Rachel Lees, Anna Borissova, Kat Petrilli, Manuela Giugliano, Denisa Clisu, Christelle Langley, Barbara J Sahakian, H Valerie Curran, Will Lawn, Anhedonia, Apathy, Pleasure, and Effort-Based Decision-Making in Adult and Adolescent Cannabis Users and Controls, International Journal of Neuropsychopharmacology, Volume 26, Issue 1, January 2023, Pages 9–19, https://doi.org/10.1093/ijnp/pyac056

There was an error in the coding of the Apathy Evaluation Scale (AES) resulting in incorrect scores being used for analyses in the original version of the manuscript. Specifically, items 6, 10, and 11 of the AES should have been reverse-coded but were not. All applicable analyses have been rerun with the correct scoring and results are detailed below. This correction does not alter the overall pattern of results, or any conclusions drawn in the original manuscript.

The corrected mean (standard deviation) score on the AES scale was 17.49 (6.84) for adolescent users, 15.78 (8.69) for adolescent controls, 10.96 (8.65) for adult users, and 10.97 (7.73) for adult controls. As in the original manuscript, scores ranged from 0 to 54 with higher scores indicating greater levels of apathy. The cut-off for clinical apathy (>18) was met by 22 (46.8%) adolescent users, 16 (32.0%) adolescent controls, 10 (18.2%) adult users, and 8 (13.6%) adult controls. The corrected mean (standard deviation) score on the AES ‘cognitive’ subscale was 8.53 (3.85) for adolescent users, 6.74 (4.02) for adolescent controls, 4.67 (4.18) for adult users, and 4.44 (3.51) for adult controls. The corrected mean (standard deviation) score on the AES ‘behavioural’ subscale was 3.45 (1.93) for adolescent users, 3.46 (2.57) for adolescent controls, 2.75 (2.40) for adult users, and 2.80 (2.40) for adult controls. The ‘emotional’ and ‘other’ subscales remained unchanged. Please see the attached figure which displays the correct means for the full AES scale and the cognitive and behavioural subscales.

As in the original manuscript, there were statistically significant correlations between AES scores and scores on the Snaith-Hamilton Pleasure Scale (*r* =.50, *p* <.001), Real Reward Pleasure task (RRPt) reward liking (*r* = -.26, *p* =.002), and Physical Effort task (PhEft) effort sensitivity (*r* =.21, *p* =.03), though the latter was not significant after correction for multiple comparisons. There were no significant correlations between AES scores and RRPt reward wanting (*r* = -.16, *p* =.07), PhEft total acceptances (*r* = -.02, *p* =.85), or PhEft reward sensitivity (*r* = -.03, *p* =.73).

Results from 2X2 analyses of covariance for the full AES and the truncated AES excluding item 4 (which was missing and imputed in all original and corrected analyses) are included below. As in the original manuscript, adolescents had significantly higher AES scores than adults, but there were no significant effects of User-Group or User-Group*Age-Group. Bayesian analyses showed substantial evidence for the null hypothesis of no difference between users and controls (BF_01_ = 5.36). Finally, there was no correlation between AES scores and days per week of cannabis use within the user-group (*r* =.18, *p* =.08).

**Table:** 

	F	df	p	η _ p _^ 2 ^
**AES**				
User-Group	0.02	1, 201	.90	<.001
Age-Group	13.92	1, 201	<.001	.065
User-Group*Age-Group	0.31	1, 201	.58	.002
BDI	88.07	1, 201	<.001	.305
RT-18	0.12	1, 201	.73	<.001
Alcohol	0.001	1, 201	.97	<.001
Cigarettes/roll-ups	0.58	1, 201	.45	.003
Illicit drugs	0.09	1, 201	.76	<.001
Maternal education	0.15	1, 201	.70	<.001
**AES truncated**				
User-Group	<.001	1, 201	.98	<.001
Age-Group	13.51	1, 201	<.001	.063
User-Group*Age-Group	0.24	1, 201	.63	.001
BDI	93.11	1, 201	<.001	.317
RT-18	0.26	1, 201	.61	.001
Alcohol	0.01	1, 201	.92	<.001
Cigarettes/roll-ups	0.63	1, 201	0.43	.003
Illicit drugs	0.06	1, 201	.80	<.001
Maternal education	0.12	1, 201	.73	.001