# Supplementary material for: Anhedonia, Apathy, Pleasure, and Effort-Based Decision-Making in Adult and Adolescent Cannabis Users and Controls
Source: Int J Neuropsychopharmacol. 2022 Aug 24;26(1):9–19. doi: 10.1093/ijnp/pyac056 (PMC9850660; doi:10.1093/ijnp/pyac056)
Supplement: pyac056_suppl_Supplementary_Material [file pyac056_suppl_supplementary_material.docx]

**Title:** Anhedonia, apathy, pleasure, and effort-based decision-making in adult and adolescent cannabis users and controls

**SUPPLEMENTARY MATERIALS**

**Authors:** Martine Skumlien MRes^1,2^, Claire Mokrysz PhD^2^, Tom P Freeman PhD^2,3^, Vincent Valton PhD^4^, Matthew B Wall PhD^2,5,6^, Michael Bloomfield PhD^7^, Rachel Lees MSc^2,3^, Anna Borissova MBBS^2,8^, Kat Petrilli MRes^2,3^, Manuela Giugliano MSc^2^, Denisa Clisu MSci^2^, Christelle Langley PhD^1,9^, Barbara J Sahakian PhD DSc^1,9^, H Valerie Curran PhD^2^, Will Lawn PhD^2,10^

**Affiliations:**

^1^Department of Psychiatry, University of Cambridge, Cambridge, UK; ^2^Clinical Psychopharmacology Unit, Clinical Educational and Health Psychology Department, University College London, London, UK; ^3^Addiction and Mental Health Group (AIM), Department of Psychology, University of Bath, Bath, UK; ^4^Institute of Cognitive Neuroscience, Division of Psychology and Language Sciences, University College London, London, UK; ^5^Invicro, London, UK.; ^6^Faculty of Medicine, Department of Metabolism, Digestion and Reproduction, Imperial College London, London, UK; ^7^Division of Psychiatry, University College London, London, UK; ^8^Institute of Psychiatry, Psychology and Neuroscience, King’s College London, London, UK; ^9^Behavioural and Clinical Neuroscience Institute, University of Cambridge, UK; ^10^National Addiction Centre, Institute of Psychiatry Psychology and Neuroscience, King’s College London, London, UK.

**Section 1. Supplemental methods**

**Table S1.** Inclusion and exclusion criteria for all participants, and specific criteria for each group.

|  | Inclusion criteria | Exclusion criteria |
| --- | --- | --- |
| All participants | - Able to come to University College London five times over the next year - Capacity to give informed consent - Normal or corrected-to-normal vision - Fluent in English | - Any illicit drug use within 48 hours of the behavioural baseline session, verified with self-report and saliva testing - Any cannabis or alcohol use within 12 hours of the behavioural baseline session, verified with self-report and saliva/breathalyser testing. - Personal history of a diagnosed psychotic episode or disorder - Any one illicit drug taken >2 days/month (averaged over last 3 months) (except laughing gas) - Use of laughing gas >1 day/week (averaged over last 3 months) - Receiving treatment for any mental health condition, including cannabis dependence, in the last month - Unwilling to give blood samples or likely to faint on blood sampling - Current daily use of a medication which is commonly psychotropic - Any mental or physical health problem judged to be problematic for the study, by a medical doctor |
| Teenage cannabis users | - Aged 16-17 years - Cannabis use at a frequency of 1-7 days/week (averaged over last 3 months) | - Age-adjusted BMI <2^nd^ percentile or >99.6^th^ percentile |
| Teenage controls | - Aged 16-17 years - Between 1 and 10 days of lifetime cannabis use ***or*** 0 days of lifetime cannabis use and at least 1 day of lifetime cigarette/roll-up use | - Age-adjusted BMI <2^nd^ percentile or >99.6^th^ percentile - Cannabis use more than once in the last 3 months before behavioural baseline session - Cannabis use in the month prior to the behavioural baseline session |
| Adult cannabis users | - Aged 26-29 years - Cannabis use at a frequency of 1-7 days/week (averaged over last 3 months) | - Before the age of 18, cannabis use at a frequency of once per week or more for a period of 3 months or more. - BMI <18.5 or BMI>34.9 |
| Adult controls | - Aged 26-29 years - Between 1 and 10 days of lifetime cannabis use ***or*** 0 days of lifetime cannabis use and at least 1 day of lifetime cigarette/roll-up use | - Cannabis use more than once in the last 3 months before behavioural baseline session - Cannabis use in the month prior to the baseline session - BMI <18.5 or BMI>34.9 |

Abbreviations: BMI, Body Mass Index.

**Questionnaire measures**

Anhedonia was assessed with the Snaith-Hamilton Pleasure Scale (SHAPS) (Snaith et al., 1995). The SHAPS consists of 14 items designed to measure the respondent’s ability to experience pleasure, e.g. “I would enjoy being with family or close friends”. The items were scored between 1 (definitely agree) and 4 (definitely disagree), thus total scores ranged between 14 and 56. Higher scores indicated higher levels of anhedonia. Apathy was assessed with the Apathy Evaluation Scale (AES), which consists of 18 items tapping cognitive, emotional, and behavioural dimensions of amotivation (Marin et al., 1991). Items were scored between 0 and 3, yielding a total score range of 0 to 54. Higher scores indicated higher levels of apathy.

**Behavioural tasks**

In the Physical Effort task (PhEft), participants performed button-presses in order to win points, which were later exchanged for chocolates or sweets to take home. Difficulty levels (20%, 40%, and 60%) and reward levels (2, 7, and 12 points) were presented at the beginning of each trial. More difficult trials required faster button pressing, and participants pressed with their little finger on their non-dominant hand. On each trial the participant could choose to accept or reject the offer, and points were only accrued if the trial was accepted and completed. The task included five trials of each difficulty and reward level combination, resulting in 45 trials in total, and took 15 minutes to complete. The order of trials was randomised.

In the RRPt participants were told to estimate how much they wanted to 30 seconds of one of their favourite songs, one piece of chocolate/candy, and a one-pound coin, on a 20 cm visual analogue scale, with anchors “do not want at all” and “intensely want”. They then received each reward in turn and were asked to rate how pleasurable they found them on a 20 cm visual analogue scale, with anchors “do not like at all” and “intensely like”. They were also asked to rate how much they generally like chocolate/sweets and the song chosen at the beginning of the task on a scale of 1 to 10.

**Covariates**

Depressive symptomatology was assessed with the Beck Depression Inventory (BDI) (Beck et al., 1996). Risk-taking was assessed with the risk-taking 18 (RT-18) questionnaire (de Haan et al., 2011). Maternal education was used as a proxy for socioeconomic status and measured dichotomously as below undergraduate degree and undergraduate degree or above. Alcohol and non-cannabis tobacco use were measured as average number of days per week of use over the past three months. Other illicit drug use was measured dichotomously as minimum monthly use of any one drug (yes/no), averaged over the past three months.

**
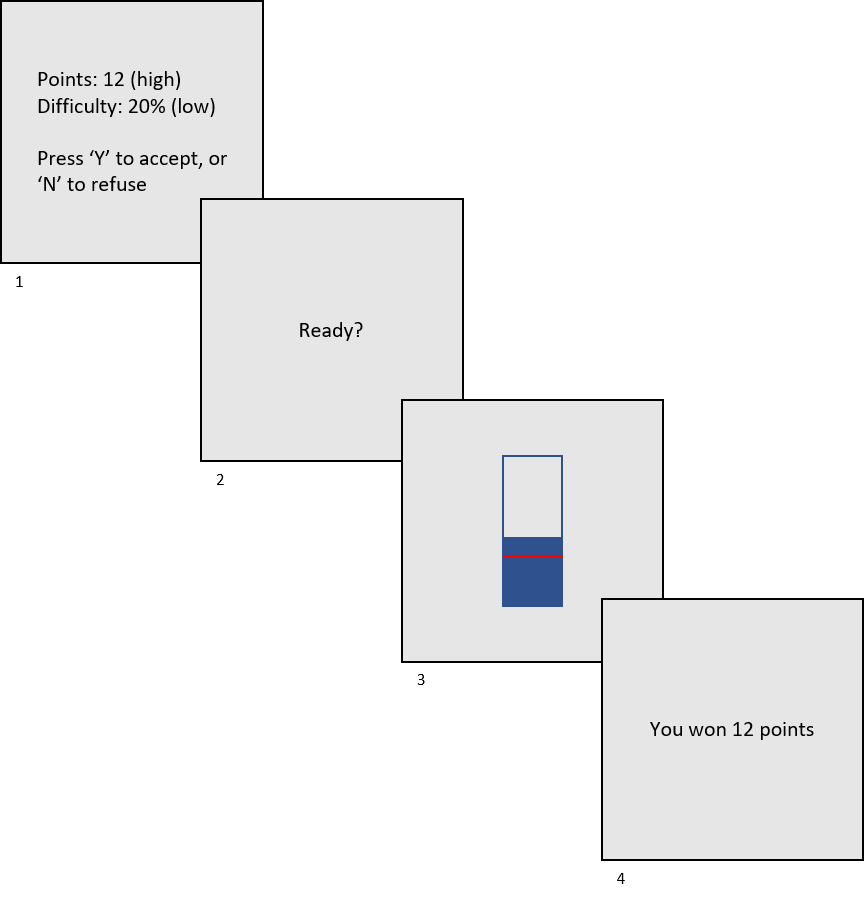
**

**Figure S1.** The Physical Effort task. High reward low effort success trial example. In stage (3), the red line indicates the difficulty/effort level. The participant has to repeatedly press the spacebar button with their little finger on their non-dominant hand to prevent the blue bar from dropping below the red line, in order to win the points.

**Table S2.** Imputation and reasons for exclusion for all measures

| **Measure** | **Total completed** | **Excluded** | **Missing values imputed** | **Total included in analyses** |
| --- | --- | --- | --- | --- |
| **Full sample** (completed baseline behavioural session) | | | | |
| SHAPS | **n=274 total**  n=76 teen users  n=71 adult users  n=63 teen controls  n=64 adult controls | Missing maternal education (n=6) | Item 3 (n=1) | **n=268**  n=75 teen users  n=68 adult users  n=62 teen controls  n=63 adult controls |
| AES | **n=215 total^a^**  n=48 teen users  n=57 adult users  n=50 teen controls  n=60 adult controls | Missing maternal education (n=4) | Item 11 (n=1)  Item 13 (n=1)  Item 16 (n=1)  Item 4 missing and imputed for all participants | **n=211**  n=47 teen users  n=55 adult users  n=50 teen controls  n=59 adult controls |
| **Subsample** (also completed baseline neuroimaging session) | | | | |
| PhEft total acceptances | **n=139 total**  n=34 teen users  n=35 adult users  n=35 teen controls  n=35 adult controls | Missing maternal education (n=2) | N/A | **n=137**  n=33 teen users  n=35 adult users  n=35 teen controls  n=34 adult controls |
| PhEft reward sensitivity | **n=139 total**  n=34 teen users  n=35 adult users  n=35 teen controls  n=35 adult controls | Reward sensitivity ≤0 (n=26)  Missing maternal education (n=1) | N/A | **n=112**  n=29 teen users  n=30 adult users  n=26 teen controls  n=27 adult controls |
| PhEft effort sensitivity | **n=139 total**  n=34 teen users  n=35 adult users  n=35 teen controls  n=35 adult controls | Effort sensitivity ≤0 (n=35)  Missing maternal education (n=1) | N/A | **n=103**  n=28 teen users  n=26 adult users  n=25 teen controls  n=24 adult controls |
| RRPt wanting & liking | **n=139 total**  n=34 teen users  n=35 adult users  n=35 teen controls  n=35 adult controls | No rating for chocolate/sweets (n=2)  Missing maternal education (n=2) | N/A | **n=135**  n=32 teen users  n=35 adult users  n=35 teen controls  n=33 adult controls |

^a^The AES was not completed by the full sample as it was added as a measure after data collection had started.

Abbreviations: AES, Apathy Evaluation Scale; PhEft, Physical Effort task; RRPt, Real Reward Pleasure task; SHAPS, Snaith-Hamilton Pleasure Scale.

**Section 2. Supplemental results**

**Table S3.** Sample characteristics for the partial sample (*n* = 137)

|  | **Adolescent users**  (*n* = 33) | **Adult users**  (*n* = 35) | **Adolescent controls**  (*n* = 35) | **Adult controls**  (*n* = 34) | **Group differences** |
| --- | --- | --- | --- | --- | --- |
| Gender  Female  Male | 16 (48%)  17 (52%) | 18 (51%)  17 (49%) | 17 (49%)  18 (51%) | 17 (50%)  17 (50%) | ns |
| Age in years | 17.17 (0.51), 16.26-17.95 | 27.79 (1.12), 26.22-29.92 | 17.16 (0.47), 16.23-17.98 | 27.43 (0.91), 26.03-29.74 | Adults > Adolescents*** |
| Ethnicity  White  Mixed  Asian  Black  Other  Prefer not to say | 23 (70%)  6 (18%)  0 (0%)  2 (6%)  2 (6%)  0 (0%) | 25 (71%)  3 (9%)  3 (9%)  3 (9%)  1 (3%)  0 (0%) | 23 (66%)  5 (14%)  4 (11%)  2 (6%)  0 (0%)  1 (3%) | 23 (68%)  0 (0%)  8 (23.5%)  2 (6%)  1 (3%)  0 (0%) |  |
| Maternal education  Below undergraduate degree  Undergraduate degree or above | 15 (45%)  18 (55%) | 15 (43%)  20 (57%) | 14 (40%)  21 (60%) | 20 (59%)  14 (41%) | ns |
| BDI | 10.55 (6.47), 1-31 | 8.46 (10.47), 0-46 | 9.43 (6.23), 0-27 | 6.88 (6.59), 0-26 | ns |
| RT-18 | 11.52 (3.42), 3-18 | 7.86 (4.03), 3-15 | 8.63 (3.97), 0-17 | 7.74 (4.58), 0-16 | Adolescent users > Adolescent controls** |
| WTAR | 112.42 (8.57), 90-127, *n* = 31 | 107.49 (10.03), 85-124 | 110.34 (9.24), 87-126 | 111.26 (10.02), 85-122 | ns |
| Alcohol use, days/week | 0.89 (0.76), 0-3.25 | 1.55 (1.33), 0-5.25 | 0.87 (0.92), 0-3.67 | 1.47 (1.23), 0-5.25 | Adults > Adolescents*** |
| Cigarette/roll-up use, days/week | 2.15 (2.74), 0-7 | 1.64 (2.77), 0-7 | 0.65 (1.80), 0-6.58 | 0.67 (1.81), 0-7 | Users > Controls ** |
| Other illicit drug use, monthly use  Yes  No | 18 (55%)  15 (45%) | 8 (23%)  27 (77%) | 1 (3%)  34 (97%) | 0 (0%)  34 (100%) | Users > Controls***  Adolescents > Adults* |

*p<.05

**p<.01

***p<.001

Abbreviations: BDI, Beck Depression Inventory; RT-18, Risk-taking 18; WTAR, Wechsler Test of Adult Reading.

Sample characteristics are displayed for *n* = 137, which is the maximum number of participants from the partial sample included in at least one analysis. For continuous data mean (SD) and range are shown. For categorical data, n (%) is shown. Group differences were investigated with 2X2 analyses of variance, independent samples *t*-tests, or chi-square tests of independence. All variables were assessed at the baseline behavioural session.

**Table S4.** Cannabis use variables for the partial sample (*n* = 137)

|  | **Adolescent users**  (*n* = 33) | **Adult users**  (*n* = 35) | **Adolescent controls**  (*n* = 35) | **Adult controls**  (*n* = 34) | **Group differences** |
| --- | --- | --- | --- | --- | --- |
| Ever use (controls) |  |  | 31 (89%) | 33 (97%) | ns |
| Number of lifetime uses (controls) |  |  | 3.63 (2.91), 0-10 | 4.71 (3.13), 0-10 | ns |
| Days/week of use (users) | 3.29 (2.15), 0.83-6.92 | 3.83 (2.10), 0.75-6.92 | N/A | N/A | ns |
|  |  |  |  |  |  |
| Number of users who most commonly use strong herbal cannabis (i.e., ‘skunk’) | 27 (82%) | 29 (83%) |  |  | ns |
| Grams used on a day of use (users) | 0.92 (0.81), 0.15-4 | 0.72 (0.84), 0.03-3.5, *n* = 34 |  |  | ns |
| Days since last use (users)^a^ | 1.82 (1.34), 0.52-5.67 | 1.75 (1.78), 0.50-7.71 |  |  | ns |
| Age of first ever use (users) | 14.71 (1.12), 12.0-16.50 | 18.05 (3.27), 13.0-24.08, *n* = 34 |  |  | Adults > Adolescents*** |
| Age of first weekly use (users) | 15.79 (1.06), 13.25-17.67 | 22.29 (2.95), 17.0-27.67 |  |  | Adults > Adolescents*** |
| CUDIT-R (users) | 15.33 (6.16), 5-27 | 11.86 (5.70), 3-26 |  |  | Adolescents > Adults* |

*p<.05

**p<.01

***p<.001

^a^3 adolescent users and 4 adult users had not used cannabis the week before the session, and therefore had missing values for this variable

Abbreviations: CUDIT-R; Cannabis Use Disorder Identification Test – Revised.

Sample characteristics are displayed for *n* = 137, which is the maximum number of participants from the partial sample included in at least one analysis. For continuous data mean (SD) and range are shown. For categorical data, n (%) is shown. Group differences were investigated with 2X2 analyses of variance, independent samples *t*-tests, or chi-square tests of independence. Days since last use was assessed at the baseline imaging session. All other variables were assessed at the baseline behavioural session.

**Table S5.** Bivariate correlations between reward processing measures

|  | SHAPS | AES | PhEft total acceptances | PhEft reward sensitivity | PhEft effort sensitivity | RRPt reward wanting |
| --- | --- | --- | --- | --- | --- | --- |
| AES | ***r* = .515,**  ***p* < .001** | *n* = 215 |  |  |  |  |
| PhEft total acceptances | *r* = .027,  *p* = .75 | *r* < .001,  *p* = .996 | *n* = 139 |  |  |  |
| PhEft reward sensitivity | *r* = .063,  *p* = .51 | *r* = .019,  *p* = .84 | ***r* = -.647,**  ***p* <.001** | *n* = 113 |  |  |
| PhEft effort sensitivity | *r* = .153,  *p* = .12 | ***r* = .210,**  ***p* = .03^a^** | ***r* = -.380,**  ***p* < .001** | *r* = .059,  *p* = .56 | *n* = 104 |  |
| RRPt reward wanting | *r* = -.125,  *p* = .15 | *r* = -.139,  *p* = .10 | *r* = -.064,  *p* = .46 | *r* = -.096,  *p* = .31 | *r* = .043,  *p* = .66 | *n* = 137 |
| RRPt reward liking | ***r* = -.288,**  ***p* < .001** | ***r* = -.244,**  ***p* = .004** | *r* = -.149,  *p* = .08 | *r* = .052,  *p* = .59 | *r* = .122,  *p* = .22 | ***r* = .519,**  ***p* < .001** |

^a^Not significant after multiple comparisons correction

Abbreviations: As in Table S2.

Multiple comparisons correction was performed using the Benjamin-Hochberg false discovery rate procedure, with a priori q < .05.

**Table S6.** Full results from the 2X2 ANCOVAs for anhedonia (SHAPS) and apathy (AES)**,** with factors User-Group, Age-Group, User-Group*Age-Group, and covariates

|  | **F** | **df** | **p** | **η_p_^2^** |
| --- | --- | --- | --- | --- |
| **SHAPS** |  |  |  |  |
| User-Group | 5.37 | 1, 258 | .02 | .020 |
| Age-Group | 17.98 | 1, 258 | <.001 | .065 |
| User-Group*Age-Group | 1.01 | 1, 258 | .32 | .004 |
| BDI | 27.85 | 1, 258 | <.001 | .097 |
| RT-18 | 2.51 | 1, 258 | .12 | .010 |
| Alcohol | 0.22 | 1, 258 | .64 | .001 |
| Cigarettes/roll-ups | 0.03 | 1, 258 | .86 | <.001 |
| Illicit drugs | 0.97 | 1, 258 | .33 | .004 |
| Maternal education | 0.13 | 1, 258 | .72 | <.001 |
| **AES** |  |  |  |  |
| User-Group | 0.05 | 1, 201 | .82 | <.001 |
| Age-Group | 13.89 | 1, 201 | <.001 | .065 |
| User-Group*Age-Group | 0.39 | 1, 201 | .54 | .002 |
| BDI | 81.28 | 1, 201 | <.001 | .288 |
| RT-18 | 4.15 | 1, 201 | .04 | .020 |
| Alcohol | 0.06 | 1, 201 | .81 | <.001 |
| Cigarettes/roll-ups | 0.002 | 1, 201 | .97 | <.001 |
| Illicit drugs | 0.19 | 1, 201 | .66 | .001 |
| Maternal education | 0.01 | 1, 201 | .94 | <.001 |
| **AES truncated**^a^ |  |  |  |  |
| User-Group | 0.06 | 1, 201 | .80 | <.001 |
| Age-Group | 13.52 | 1, 201 | <.001 | .063 |
| User-Group*Age-Group | 0.38 | 1, 201 | .54 | .002 |
| BDI | 83.56 | 1, 201 | <.001 | .294 |
| RT-18 | 3.81 | 1, 201 | .052 | .019 |
| Alcohol | 0.07 | 1, 201 | .80 | <.001 |
| Cigarettes/roll-ups | 0.003 | 1, 201 | .96 | <.001 |
| Illicit drugs | 0.26 | 1, 201 | .61 | .001 |
| Maternal education | 0.001 | 1, 201 | .97 | <.001 |

^a^Item 4 not imputed and total score is based on 17 items.

Abbreviations: AES, Apathy Evaluation Scale; ANCOVA, analysis of covariance; BDI, Beck Depression Inventory; RT-18, Risk-taking 18; SHAPS, Snaith-Hamilton Pleasure Scale.

**Clinical anhedonia and apathy**

The original criteria proposed by Snaith et al. (1995) suggests that clinically relevant anhedonia is indicated if the respondent disagrees with more than two statements on the Snaith-Hamilton Pleasure Scale. Scores meeting the clinical cut-off for anhedonia were found in 20% of adolescent users (*n*=15), 29% of adolescent controls (*n*=18), 10% of adult users (*n*=7), and 11% of adult controls (*n*=7). For the Apathy Evaluation Scale scores above 18 are considered clinically relevant (Santangelo et al., 2014). Scores meeting the clinical cut-off for apathy were found in 68% of adolescent users (*n*=32), 58% of adolescent controls (*n*=29), 29% of adult users (*n*=16), and 36% of adult controls (*n*=21).

**
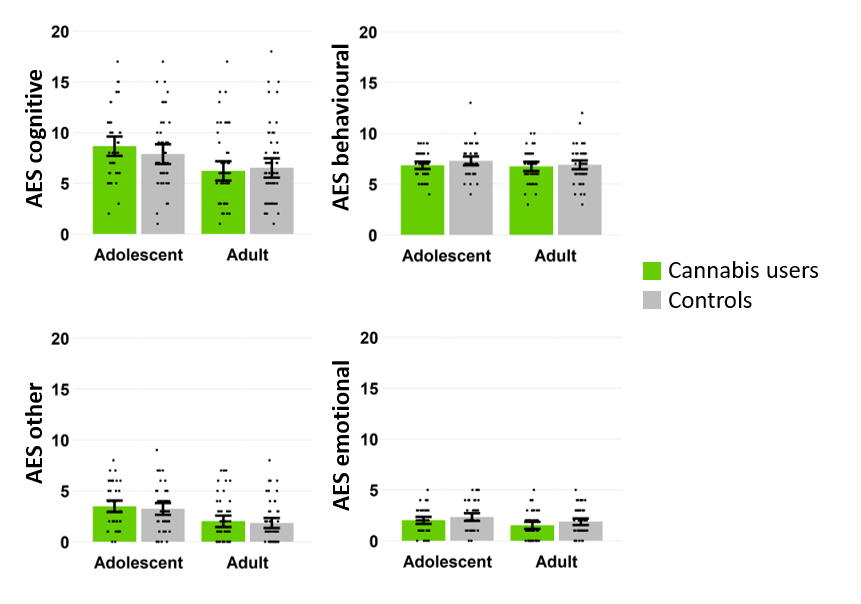
**

**Figure S2.** Group differences in Apathy Evaluation Scale (AES) subscales. Bars represent means with dots indicating individual values, and error bars represent standard errors. Higher scores indicate higher levels of apathy.

**Table S7.** Full results from the 2X2 ANCOVAs for outcomes on the Physical Effort task, with factors User-Group, Age-Group, User-Group*Age-Group, and covariates

|  | **F** | **df** | **p** | **η_p_^2^** |
| --- | --- | --- | --- | --- |
| **Total acceptances** |  |  |  |  |
| User-Group | 0.42 | 1, 127 | .52 | .003 |
| Age-Group | 0.03 | 1, 127 | .88 | <.001 |
| User-Group*Age-Group | 2.46 | 1, 127 | .12 | .019 |
| BDI | 6.09 | 1, 127 | .02 | .046 |
| RT-18 | 1.89 | 1, 127 | .17 | .015 |
| Alcohol | 0.69 | 1, 127 | .41 | .005 |
| Cigarettes/roll-ups | 0.03 | 1, 127 | .86 | <.001 |
| Illicit drugs | 0.19 | 1, 127 | .66 | .001 |
| Maternal education | 0.57 | 1, 127 | .45 | .004 |
| **Reward sensitivity** |  |  |  |  |
| User-Group | 1.48 | 1, 102 | .23 | .014 |
| Age-Group | 0.02 | 1, 102 | .90 | <.001 |
| User-Group*Age-Group | 0.01 | 1, 102 | .93 | <.001 |
| BDI | 4.20 | 1, 102 | .04 | .040 |
| RT-18 | 0.17 | 1, 102 | .68 | .002 |
| Alcohol | 0.05 | 1, 102 | .83 | <.001 |
| Cigarettes/roll-ups | 1.68 | 1, 102 | .20 | .016 |
| Illicit drugs | 2.30 | 1, 102 | .13 | .022 |
| Maternal education | 0.91 | 1, 102 | .34 | .009 |
| **Effort sensitivity** |  |  |  |  |
| User-Group | 1.66 | 1, 93 | .20 | .017 |
| Age-Group | 0.91 | 1, 93 | .34 | .010 |
| User-Group*Age-Group | 0.09 | 1, 93 | .77 | .001 |
| BDI | 0.95 | 1, 93 | .33 | .010 |
| RT-18 | 0.25 | 1, 93 | .62 | .003 |
| Alcohol | 3.54 | 1, 93 | .06 | .037 |
| Cigarettes/roll-ups | 0.80 | 1, 93 | .37 | .009 |
| Illicit drugs | 0.62 | 1, 93 | .43 | .007 |
| Maternal education | 1.15 | 1, 93 | .29 | .012 |

Abbreviations: As in Table S6.

**Table S8.** Full results from the 2X2 ANCOVAs for reward wanting and reward liking on the Real Reward Pleasure task, with factors User-Group, Age-Group, User-Group*Age-Group, and covariates

|  | **F** | **df** | **p** | **η_p_^2^** |
| --- | --- | --- | --- | --- |
| **Reward wanting** |  |  |  |  |
| User-Group | 2.10 | 1, 125 | .15 | .017 |
| Age-Group | 1.05 | 1, 125 | .31 | .008 |
| User-Group*Age-Group | 0.53 | 1, 125 | .47 | .004 |
| BDI | 2.18 | 1, 125 | .14 | .017 |
| RT-18 | 1.14 | 1, 125 | .29 | .009 |
| Alcohol | 0.30 | 1, 125 | .58 | .002 |
| Cigarettes/roll-ups | 0.29 | 1, 125 | .59 | .002 |
| Illicit drugs | 1.75 | 1, 125 | .19 | .014 |
| Maternal education | 0.09 | 1, 125 | .77 | <.001 |
| **Reward liking** |  |  |  |  |
| User-Group | 1.02 | 1, 125 | .32 | .008 |
| Age-Group | 0.94 | 1, 125 | .34 | .007 |
| User-Group*Age-Group | 1.85 | 1, 125 | .18 | .015 |
| BDI | 8.07 | 1, 125 | .005 | .061 |
| RT-18 | 3.91 | 1, 125 | .05 | .030 |
| Alcohol | 1.45 | 1, 125 | .23 | .011 |
| Cigarettes/roll-ups | 0.04 | 1, 125 | .84 | <.001 |
| Illicit drugs | 2.44 | 1, 125 | .12 | .019 |
| Maternal education | 0.001 | 1, 125 | .97 | <.001 |

Abbreviations: As in Table S6.

**Table S9.** Real Reward Pleasure task means and standard deviations for individual reward types in each group

|  | **Adolescent users**  (*n* = 32) | **Adolescent controls**  (*n* = 35) | **Adult users**  (*n* = 35) | **Adult controls**  (*n* = 33) |
| --- | --- | --- | --- | --- |
| General liking of chocolate/sweets | 7.59 (1.50) | 7.40 (1.72) | 7.81 (1.88) | 8.18 (1.45) |
| Liking of song chosen | 9.50 (0.72)^a^ | 9.39 (0.75)^a^ | 9.47 (0.81) | 9.45 (0.62)^a^ |
| **Want reward,** mean (SD) | 12.65 (2.70) | 11.73 (3.21) | 12.93 (2.70) | 12.68 (2.93) |
| Want chocolate/sweets | 11.29 (4.61) | 11.48 (4.45) | 13.50 (4.51) | 13.25 (3.39) |
| Want music | 13.62 (3.65) | 10.21 (4.28) | 11.98 (4.45) | 11.14 (5.00) |
| Want one-pound coin | 13.03 (3.82) | 13.49 (4.08) | 13.31 (4.59) | 13.65 (4.80) |
| **Like reward,** mean (SD) | 14.37 (2.45) | 13.51 (2.96) | 14.18 (3.33) | 14.73 (2.87) |
| Like chocolate/sweets | 15.01 (2.95) | 14.31 (3.64) | 14.78 (4.54) | 15.95 (3.07) |
| Like music | 16.73 (3.33) | 14.98 (3.38) | 16.26 (4.48) | 16.89 (3.50) |
| Like one-pound coin | 11.38 (4.31) | 11.24 (4.36) | 11.49 (5.67) | 11.36 (4.53) |

^a^*n*=1 missing adolescent user, *n*=3 missing adolescent controls, *n*=2 missing adult controls

General liking of chocolates/sweets and liking of song chosen were rated on a scale of 1 to 10.

**References**

Beck AT, Steer RA, Brown GK (1996) Beck depression inventory-II. San Antonio, Texas: The Psychological Corporation.

de Haan L, Kuipers E, Kuerten Y, van Laar M, Olivier B, Verster JC (2011) The RT-18: a new screening tool to assess young adult risk-taking behavior. Int J Gen Med 4:575-584.

Marin RS, Biedrzycki RC, Firinciogullari S (1991) Reliability and validity of the Apathy Evaluation Scale. Psychiatry Res 38:143-162.

Santangelo G, Barone P, Cuoco S, Raimo S, Pezzella D, Picillo M, Erro R, Moccia M, Pellecchia MT, Amboni M, Santangelo F, Grossi D, Trojano L, Vitale C (2014) Apathy in untreated, de novo patients with Parkinson's disease: validation study of Apathy Evaluation Scale. J Neurol 261:2319-2328.

Snaith RP, Hamilton M, Morley S, Humayan A, Hargreaves D, Trigwell P (1995) A scale for the assessment of hedonic tone the Snaith-Hamilton Pleasure Scale. Br J Psychiatry 167:99-103.
